# Notes on Prevention and Early Treatment of Mental Disease1Read before the Medical Society of the County of Erie.

**Published:** 1896-10

**Authors:** Arthur W. Hurd

**Affiliations:** Superintendent Buffalo State Hospital


### NOTES ON PREVENTION AND EARLY TREATMENT OF MENTAL DISEASE.[1]

By ARTHUR W. HURD, A. M., M. D., superintendent Buffalo State Hospital.

THE question of prevention involves a brief consideration of the periods and epochs in which nervous and mental disease is most likely to appear in the human organism, especially in those whose heredity is bad. Attention to this point, when we have under consideration patients with an ancestry afflicted with epilepsy, insanity, alcoholism and the like, may lead to such measures as will avoid an attack of mental disease at some of the critical periods.

The period of most rapid growth of special sense-education, of coördination and of speech, is from birth to seven years, and in this, as we shall see later, many of the neuroses make their appearance. To Dr. Clouston much is due for valuable work in this field.

It is interesting to note that perfection of form and size is not always coincident with maturity of function, as, for instance, the brain, up to seven years of age, grows nearly to its later full size, but it does not reach its perfection of function until about twenty-five. It would seem that in coming to perfection it is not the quantity so much as quality which is, in the years of adolescence, perfected. The blood corpuscles and blood vessels in their struc-

1. Read before the Medical Society of the County of Erie.

ture are, probably, at birth as perfect as in later years, but of the brain and nervous system we find that many years, almost twenty-five, are needed to complete their perfection of function.

It is also interesting to note that it is in these tissues (which are latest developed) that diseases which may be traced to a bad heredity are prone to make themselves evident. It is during the long period of gradual coming to perfection of the nerve cell, as has been said, that hereditary influences for good or evil come most into visible play.

Nature provides different periods when the different organs and functions of the body shall come to maturity, and has retained a regular and healthful sequence in which the powers of the human being shall be developed. It is often unfortunate that certain capacities or powers are developed outside of their regular order or sequence. The stimulation and development of the emotional or sexual or imaginative qualities before the bringing to full maturity of the muscular system, has often resulted in ill-balanced youths, in precocity of intellect, in nervous disease and in premature decay. If we can be on our guard against the development of certain functional neuroses at different periods of life in children who have a history of nervous troubles or of insanity in their ancestry, we may do something in prevention.

First, we have the formative and embryonic state. In this stage, preceding birth, it is when hair-lip, deformed and cleft palates, spinabifida, club-feet, malformation of organs, cephalic deformities and the like are found.

The second period of which we speak is from birth up to seven years of age. It is the period of the most brain growth during life. It is the period when the special senses are being educated, when speech is being acquired and when the infants are learning to coördinate muscular movements, and in this period it is that we are most to expect the development of rickets, of night terrors, convulsions of teething, infantile paralysis, epilepsy, general backwardness of speech, stammering, squinting of the eyes, a strumous condition, ophthalmia, various forms of skin diseases, liability from nervous causes to sudden rises of temperature and great susceptibility to attacks of all zymotic diseases, delirium at night with low temperatures, tubercular meningitis, hydrocephalus and the like.

From seven to thirteen years of age may be called the period of coördination of motion and emotion, when there is going on, along with the development of the body, a development of mental qualities as well, and in this period we are apt to have developed chorea, epilepsy, asthma, sleep walking, headache and eye troubles.

From thirteen to twenty-five years we have the period of puberty and adolescence, and in this also we find epilepsy developed ; asthma, chlorosis, mental defects, hysteria, migraine, adolescent insanity, tendency to outbursts of temper, perversions of the moral sense, perverted sexual instincts, arrested development —apparently both bodily and mental—acne, and possibly phthisis. Sometimes the lower or more animal elements of the individual nature become fairly well developed, and then stops the higher cerebral elaboration which marks the higher intelligence of maturity, and we have as a result the shiftless "ne'er-do-wells," the "grown-up children," who are impulsive, quick in temper, lacking in judgment, the defectives of the world, who require the supervision of their stronger brethren. It is this class which largely makes up, it is supposed, the "submerged tenth," to use a term coined, I believe, by General Booth.

The power of attention, memory, reason, volition, and the like, may vary within reasonable limits, but nature sets a reasonable sequence in which they shall be developed. The boy who develops a great capacity for solving amazing problems in mathematics, the prodigy of intellectual activity, is one-sidedly developed, and is apt to wear out his cerebral force long before the time when it should be at its highest point of development.

It is essential for the family physician who has such subjects under his care, to maintain a wise supervision of the education and development of such children, for in these cases of bad heredity often a judicious course of life and training will avoid an attack of nervous disease, or even insanity, and this is of much more moment than the treatment of it after it has been established. As has been said, when the period of adolescence is reached "we hear from our grandparents ;" in other words, it is in this period when defects of heredity are most likely to crop out.

There are insanities of adolescence, of pregnancy, lactation and senility, all of which are to be looked for by the family physician in subjects predisposed thereto, and in many cases avoidance can be obtained, for being "forewarned is forearmed."

We must remember that in adolescence the condition is one of mental and bodily development, of extension, of growth and evolution ; whereas in senility there is involution, decadence and diminution of activities, both mental and physical. In the former we must avoid excesses, errors of development, undue strains, either in school or in responsibilities, and in the other the energies must be directed to keeping up the waning powers of life at as great a level of effectiveness as possible.

In this connection the recent studies in psychology are of interest, and when fully worked out should be of great practical benefit. To Prof. G. Stanley Hall we must acknowledge our indebtedness for his great labors in the field and for suggestions made use of in this connection. Many of the problems of the new psychology, so-called, seem trivial and their practical utility obscure for the present, but no one can tell where or when the application of scientific facts accumulated may possibly be made.

The child is extremely active as regards his muscular system, as every observer knows. There seems to be, as proved by recent psychological experiments, a direct connection between muscular action and thought, and the curbing of muscular activity in childhood is to be deprecated. Does anyone doubt that muscular action accompanies mental exertion, it is only necessary to see a roomful of children put to writing a certain form or article. The mental exertion evidences itself in cramped fingers, crooked postures, scowling, placing the tongue in one cheek, screwing up the mouth, twisting the limbs, and the like, and as many different apparently unnecessary muscular movements as there are children in the room. This condition lessens with the gradual growth and discipline of the brain, but even in the most cultivated oftentimes there still linger "habit-spasms" of speech, gesture, articulation, and the like, which persist often into adult life.

And in this connection it is well to note, as has been pointed out by Professor Hall and others, that intellectual work is not in itself harmful. With the development of civilisation it seems that there is not now a shortening of the average duration of existence, but perhaps rather a lengthening, and we see, too, an extension of the period of intellectual absorption and of study, the period of so-called education.

Formerly a child went to school at five or seven years of age and ceased early, perhaps from sixteen to eighteen years of age. Now the period of education in schools is extended in both directions—downward by the Kindergarten and upward by the universities—so that under modern conditions many children commence education at two or three years and extend it to twenty-five or thirty years of age in their university or post-graduate course. And this extended period of preparation seems not to result in deteriorated health if the studies of childhood and youth are directed in proper channels, and if the physical condition in the meantime is carefully preserved. Ordinarily the brain, if unexposed to undue influences and inheriting a sound organism, is able to withstand the excessive evolution which occurs in the period of adolescence, but those with a neurotic or insane heredity often are unable to withstand this strain, and consequently at this period show eccentricities, excessive activity, motor, muscular, and the like, and break down.

It should be a matter of interest to the physician that these cases should pass in safety this period. To such patients the rush and competition of the public school, where all grades of intellect, all brains, no matter how varied in strength or how diverse in capacities or powers of resistance, where all are placed at the same tasks with the same struggle for supremacy, is probably one of the poorest methods of development. Their education should be special and individual and not general. As a rule, the memorising of facts or other purely intellectual processes involved in grasping abstract ideas, together with a sedentary life and the confinement in doors, is not the ideal mode of training. The development which comes from manual exercise, from the study of natural history in the fields, from the study of mechanics in the workshop, is best suited to develop symmetrically the different parts of the mental organism.

Could the physician always see patients in the early stages of illnesses which lead later to different forms of insanity, it is probable that many cases could be prevented. No one can tell to what lengths of mental disturbance an apparently slight illness may lead, and a slight review of some of the usual bodily conditions which precede and accompany mental disturbances may be, while commonplace, yet useful.

Among the first of the conditions which frequently precede mental disturbances I will mention disorders of sleep. This is a well-known fact, but I mention it because it is brought home to us constantly and is a condition which, if not remedied, always leads to serious conditions, if not actual insanity. And by disorders of sleep I do not restrict the term to mere insomnia, but also to include other unnatural conditions, such as carrying business worry through the night, being unable to throw off the burdens of the day when rest is sought; uneasy, restless sleep, talking in one's sleep, a sense of exhaustion in the morning and a recurrence of unpleasant dreams. All this results in a condition of nerve tire and exhaustion, in which the refreshing buoyancy of life is lost.

One of the recent writers on psychology has said that from the surplusage of energy, such as is felt in the morning after a refreshing night's sleep, is built the progress of the world; that much of our vitality goes to support life, to maintain one's position, to acquire food and the absolute necessities of life; but that it is from this elasticity of spirits in excess, this surplusage of energy, that we obtain our accumulated store, if we may so speak, from which springs progress and the development of new ideas. And while this idea may be somewhat fanciful, it is, however, a useful one to bear in mind. For psychology is teaching us, among other things, that the material aim of bodily health is happiness for the individual. A more close laboratory study of the connection between mind and body reveals to us how much of disease is dependent upon mental impressions, and conversely how much of health also.

From the standpoint of the psychologist, happiness is the foundation of perfect functioning of the different organs in normal unity. It is unnecessary to illustrate this by mentioning the derangement of digestion in those who are worried by business matters, the interference of the function of the liver by those passing through great anxiety, the development of cancer in the stomach by those who have great mental worry, and it is this fact, however much we may overlook it, which lies at the foundation of the real benefit, which sometimes comes from the mental impressions of miracle cures, faith cure and the like; the real impression on the function of organs which the mental state determines; but it is in functional troubles, as would be expected, that these results are most evident, rather than in organic disease.

And the converse of this is widely and universally true, that the condition of a patient's organism reacts upon his mental condition as well, and the physical basis of mental disease must not be lost sight of. In the treatment of insomnia, the resort to drugs should be the last method adopted. There are many in which hygienic measures, such as regulation of the diet, massage, exercise, removal of unfavorable conditions and the like will bring about relief without resort to hypnotics.

Another set of disorders which frequently precede mental disturbance are disorders of digestion, and this consists, not merely in constipation, but in failure of absorption, failure of appetite, failure of assimilation, hypersecretion of stomach fluids and the like, and this brings to our notice the subject of auto-infection, which is claiming so much attention of late. It is, unfortunately, too often common to see a condition of maniacal excitement existing, in which the patient has been deluged with bromides and other sedatives, in which the skin is hot and dry, the breath offensive, tongue coated and the bowels extremely constipated. In these cases the change that takes place upon restoring the functions of the skin, liver and bowels, is often remarkable.

It is my belief that oftentimes at the beginning of an attack of mental disturbance, attention to the condition of the bowels and skin, by means of hot baths, massage, mineral waters, or even purgatives, together with a light assimilable diet, will render an attack much less acute and much less prolonged in duration.

The subject of auto-infection is one which has but recently been studied at all thoroughly, but which promises much, and treatment directed along these lines has already accomplished a great deal.

Another form of early mental disturbance is that seen in those who have been compelled to work for long periods of time without relaxation, and these cases present symptoms of mental confusion, loss of power of application and attention. The clerk is unable to concentrate his attention so vigorously on his duties, the accountant is unable to add his figures, the lawyer and business man are unable to accomplish the same amount of work in the same time as heretofore, without constant spurring and inciting his nervous system to an exhausting pitch of intense activity. These soon become cases of cerebral exhaustion, which may eventually result in profound melancholia, or remain simple cases of neurasthenia. The tendency in this form of trouble is to make rest imperative by rendering impossible the power to work, and thus bringing about conditions which lead to restoration.

These are the cases in which the physician should interfere, and prescribe immediately an early rest, before the stage of cerebral exhaustion has been reached.

Among other prodromata of mental disturbance are numerous symptoms of cerebral tire, such as worry over trifles, magnifying trifles, great irritability, lack of calm self-control and the equipoise which should characterise the individual in health, aversion to family, unusual outbursts of temper, fatigue and the like. In these cases, as in the others, there should be distinguished the difference between fatigue and the sensation of fatigue. In some forms of disease, notably in paresis and in acute mania, there is apparently no fatigue felt. In the normal human being there is a natural and healthy fatigue, but when the nervous system has been imposed upon, even to the point of breaking down, the sense of fatigue may be lost, and this distinction between fatigue and the sensation of fatigue is an important one.

A patient says, after an hour's sleep, "I have slept five hours. I am completely refreshed, and I am able to do more work than ever in my life," while at the same time his emaciating frame and his sunken eyes all tell of the real fatigue, which is not felt by him.

It is unnecessary to mention the treatment of all the physical ailments which by inducing general ill-health may cause failure of cerebral nutrition and consequent mental symptoms. The rheumatic, the lithemic, the dyspeptic and cardiac cases all require treatment appropriate to their special conditions and the building up of their general health to avert threatened mental break-down.

Aside from the proper medical treatment necessary to all these cases, there are certain measures which are worth consideration, and which are applicable to nearly all. By these I mean removal from home influences and former surroundings and the substitution of different occupations and companions. In cases of cerebral exhaustion, of beginning melancholia, with depressing delusions, removal from home and family, and the like, is of great benefit. This does not mean travel, but it means a change to a quiet, restful place, free, as far as possible, from the conditions of annoyance and irritation which have prevailed, to the care of some kind, firm companion, who will lead, direct, divert and control, without a show of authority or irritation.

This one measure alone is worth more than all others, to my mind, in incipient mental disease. And it is one that can be tried by many patients, though not by all. The financial condition of many preclude it. When that condition exists, or when a patient of the wealthier class becomes worse and it is dangerous for him to remain at large, of course hospital treatment must be resorted to ; but in most of the patients of the well-to-do class, who come largely under the care of the family physician in the stage preceding and during actual mental aberration, this measure is extremely valuable and useful, if carried out under proper medical supervision and proper nursing. The conditions of travel as such, are not, to my way of thinking, of as great value in mental disease as is generally supposed. In certain forms of dementia it may tend to interest or arouse and to incite sluggish mental action, but in cases of acute mania or acute melancholia, travel, new sights and scenes only add to the excitement in the one case, and afford points of fixation for depressing delusions and mental pain in the other.

